# Glucocorticoid dynamics predict susceptibility and resilience to traumatic stress in male and female rats

**DOI:** 10.3389/fncel.2026.1880559

**Published:** 2026-10-09

**Authors:** Emily A. Devine, James B. Chambers, Taylorae Dunn, Ava Musarra, Sophie Porter, Christine E. Moore, Marissa Smail, Nawshaba Nawreen, Ben A. Packard, James P. Herman

**Affiliations:** 1 Department of Pharmacology, Physiology, and Neurobiology, University of Cincinnati, Cincinnati, OH, United States; 2 Neuroscience Graduate Program, University of Cincinnati, Cincinnati, OH, United States; 3 Department of Psychology, Ohio State University, Columbus, OH, United States; 4 Eli Lilly and Company, Indianapolis, IN, United States; 5 Veterans Affairs Medical Center, Cincinnati, OH, United States

**Keywords:** anxiety-related behavior, corticosterone, fear conditioning, HPA-axis, sex differences

## Abstract

**Introduction:**

The mammalian stress response relies on tightly regulated HPA axis signaling, and disruptions in glucocorticoid feedback are implicated in stress-related disorders such as PTSD. Although many individuals experience trauma, only some develop pathology, suggesting that individual differences in glucocorticoid responsivity may help predict susceptibility or resilience. Here, we examined whether corticosterone (CORT) responses to a mild stressor predict behavioral outcomes following single-prolonged stress (SPS) in male and female rats.

**Methods:**

Mild stress reactivity was quantified using area-under-the-curve (AUC) CORT responses to light-dark box exposure, after which animals underwent SPS, behavioral assays, and post-reinstatement cFos immunohistochemical analysis.

**Results:**

Females exhibited SPS-induced reductions in basal AM CORT and displayed a robust traumatic stress-related phenotype in which high CORT responders displayed increased anxiety-like behavior, impaired fear extinction, and enhanced reinstatement. Correlational and permutation analyses revealed a consistent pattern in SPS-exposed females in which lower pre-stress basal AM CORT and higher mild stress-induced CORT (AUC) predicted increased fear expression, indicating a dissociation between resting and stress-evoked glucocorticoid signals. In males, mild stress reactivity did not predict SPS-related outcomes; however, reduced pre-stress basal AM CORT in control males predicted increased fear expression, suggesting sex-specific differences in the potential predictive value of CORT dynamics. SPS and CORT responder status also differentially modulated cFos expression across stress-related brain regions, including the PVN, infralimbic cortex, and amygdala in both males and females.

**Conclusion:**

These findings demonstrate that mild stress reactivity and basal glucocorticoid dynamics can predict trauma-related phenotypes in a sex-specific manner, identifying them as a potential biomarker of PTSD susceptibility in females.

## Introduction

The mammalian stress response is a critical adaptive function, in which a complex sequence of physiological mechanisms works to enhance survival and restore homeostasis in the face of a challenge (Herman et al., 2016; Myers et al., 2012; Ulrich-Lai and Herman, 2009). A major component of this stress response involves the hypothalamo-pituitary-adrenocortical (HPA) axis, which is activated upon detection of a real or perceived threat (Herman et al., 2016; Myers et al., 2012; Ulrich-Lai and Herman, 2009). The secretion of glucocorticoids facilitates the redistribution of energy stores to ensure the necessary resources are available to address the predicted or present stressor (Herman et al., 2016; Myers et al., 2012, 2014; Ulrich-Lai and Herman, 2009). As the stressor subsides, glucocorticoids restore homeostasis by exerting negative feedback on the HPA axis via binding to glucocorticoid and mineralocorticoid receptors in key brain regions, thus inhibiting further hormone release (Herman et al., 2016; Herman et al., 2020; Myers et al., 2012, 2014; Ulrich-Lai and Herman, 2009).

Under normal conditions, acute activation of the stress response is tightly regulated via glucocorticoid negative feedback, often resulting in enhanced sensory-motor performance and improved cognitive functions (Herman et al., 2016, 2020; Myers et al., 2012; Su et al., 2025; Ulrich-Lai and Herman, 2009). However, exposure to a particularly intense, repeated, or prolonged stressor can lead to glucocorticoid signaling dysfunction and contribute to the development of stress-related diseases such as post-traumatic stress disorder (PTSD) (de Kloet, 2022; Dunlop and Wong, 2019; Göver and Slezak, 2024; Jones and Moller, 2011). It is thought that trait or state differences in glucocorticoid feedback sensitivity may be related to risk for or manifestation of PTSD-related behavioral dysfunction (Dunlop and Wong, 2019; Göver and Slezak, 2024; Jones and Moller, 2011). The resulting imbalance impairs the capacity to appropriately regulate arousal, immune response, and emotional processing (de Kloet, 2022; Dunlop and Wong, 2019; Göver and Slezak, 2024; Jones and Moller, 2011).

While many individuals experience trauma, only a portion go on to develop PTSD. Acquiring deeper insight into the variables contributing to PTSD susceptibility is crucial for improved identification and treatment of at-risk individuals. Although numerous factors can contribute to an individual’s susceptibility or resilience, previous work suggests a connection between glucocorticoid levels and PTSD symptomology (de Kloet, 2022; Dunlop and Wong, 2019; Göver and Slezak, 2024; Jones and Moller, 2011). Human studies have found correlations between pre- and post-stress cortisol levels and PTSD development (Galatzer-Levy et al., 2014; Lindauer et al., 2006; Song et al., 2008; Steudte-Schmiedgen et al., 2015; Yehuda et al., 1998) and symptom severity, while rodent studies indicate similar findings between corticosterone (CORT) and PTSD-like behavioral deficits (Belda et al., 2020; Cohen et al., 2006; Daws et al., 2017; Skórzewska et al., 2020). Notably, most of these studies were only performed in males. Additionally, it is still unknown whether stress reactivity plays a role in the development of trauma resilience or susceptibility.

In the present study, we aim to determine whether the magnitude of the CORT response to a mild stressor can predict behavioral outcomes after a later exposure to single-prolonged stress (SPS), a rodent model of traumatic stress (second hit). To do so, we exposed male and female Sprague-Dawley rats to a mild stressor (light-dark box) and collected plasma CORT across 4 time points, followed 3 weeks later by exposure to single-prolonged stress and subsequent behavioral testing. Our results suggest that high CORT secretion following mild stress can predict trauma-related behavioral phenotypes in females, suggesting a sex-dependent relationship between mild stress reactivity and risk for trauma-related pathology.

## Materials and methods

### Subjects

Young adult male (250–275 g, ∼8–9 weeks old) and female (225–250 g, ∼11 weeks old) Sprague-Dawley rats were purchased from Envigo. All animals were pair-housed in translucent polycarbonate shoebox cages with corncob bedding and left undisturbed for 1 week prior to any manipulations, with food and water available *ad libitum*. Animals exposed to SPS were single-housed immediately following said exposure. The colony room was temperature and humidity-controlled and maintained on a 12-h light-dark cycle (06:00 h lights on, 18:00 h lights off). All experiments complied with the National Institutes of Health Guidelines for the Care and Use of Animals and were approved by the Cincinnati Veterans’ Administration Medical Institutional Animal Care and Use Committee.

### Experimental design

For this experiment, male and female rats were run in separate cohorts, 3 months apart. An identical experimental design and timeline was used for both cohorts. To address whether CORT response to a mild stressor can predict post-SPS behavioral outcomes, rats were weight-matched and assigned to control or SPS groups (*n* = 20/group), and basal AM and PM CORT measurements were taken to establish each animal’s baseline levels. Three days later, all animals were exposed to mild stress via a light-dark (LD) box, and CORT was measured 15, 30, 60, and 120 min post-LD box exposure. These measurements were then used to calculate the area under the curve (AUC). Three weeks later, animals assigned to the SPS group were exposed to the SPS paradigm, while the control (CTL) animals remained in their home cages. Additional basal AM CORT measurements were taken at 1 and 7 days post-SPS.

Behavioral assessments began 1 week following SPS. To minimize the potential influence of prior testing on subsequent behavioral performance, a waiting period of 3–7 days was scheduled between each behavioral assay and assays were run in order from lowest to highest anticipated stress: (open field/novel object recognition → social interaction → elevated plus maze → fear conditioning). Animals were euthanized 90 min after fear conditioning reinstatement. In addition to behavioral testing, immunohistochemistry was utilized to measure cFos in prefrontal cortex and amygdala subregions, and the paraventricular nucleus of the hypothalamus. Once the experiment was complete, the upper and lower quartiles of the post-LD box CORT AUC measurements were determined for each group (CTL and SPS). These upper and lower quartiles were then used to subdivide each group into high (upper quartile) and low (lower quartile) CORT responders, as they displayed high or low CORT levels in response to the mild stress of the LD-box. As a result, data consisted of four groups total (*n* = 5/group). To support our findings among the high and low CORT responders, Pearson’s correlation calculations were performed using the data from the original 20 CTL and 20 SPS animals. Please see Figure 1 for a visual representation of the experimental timeline.

**FIGURE 1:**
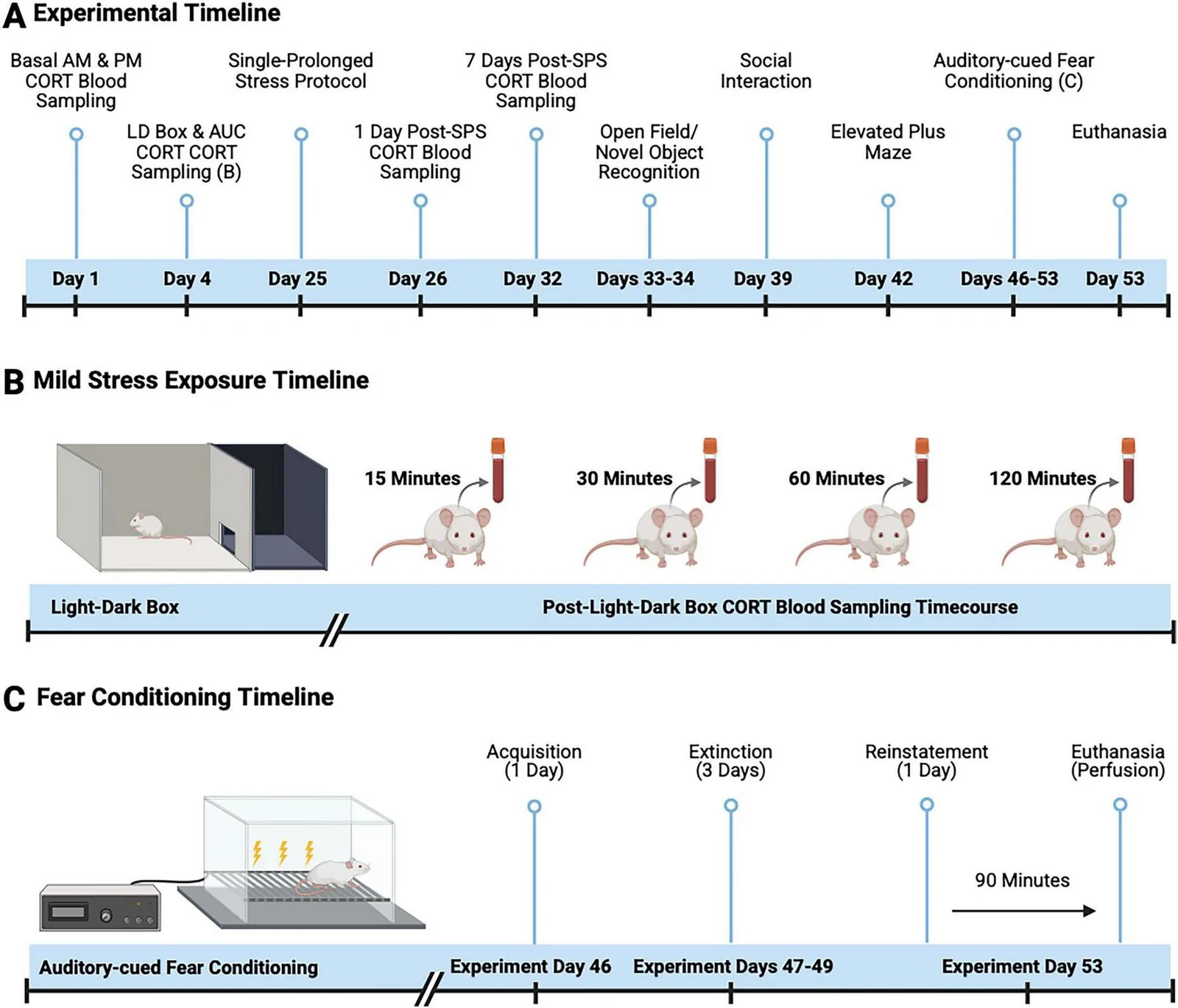
Experimental timeline. **(A)** Overview of experimental timeline. This timeline was used for both male and female cohorts. **(B)** Timeline depicting mild stress exposure via light-dark box and subsequent CORT blood sampling. **(C)** Timeline depicting fear conditioning protocol and subsequent euthanasia. Created in BioRender. Devine, E. (2026) https://core.local.biorender.dev/api/short-link/7c89jeo.

### HPA-axis assessment

Blood was collected from tail bleeds via tail tip sectioning for all CORT analyses, as previously described (Vahl et al., 2005). Blood was collected between 8 and 11 AM and 1 and 4 PM for basal AM and PM CORT measurements, respectively. Additionally, blood was collected at 15, 30, 60, and 120 min post-LD box exposure to calculate the CORT AUC.

### Corticosterone ELISA

Blood samples were centrifuged at 4 °C and 6,000 × g for 15 min, and plasma was collected and stored at −20 °C until assay preparation. Plasma CORT concentrations were determined using the Enzo Life Sciences Corticosterone ELISA kit. All samples were run in duplicate and coefficients of variations for each duplicate were under 5%.

### Single-prolonged stress

Single-prolonged stress was conducted in accordance with prior published procedures (Cotella et al., 2023; Smail et al., 2025). Rats were placed in a plastic restrainer (6.4 cm in diameter for smaller rats, 7 cm in diameter for larger rats) for 2 h, followed immediately by a 20-min group swim (*n* = 6–8 animals per tub, 45 cm in diameter, water temperature 25 ± 1 °C). Following a 10-min rest, rats were placed in pairs into a glass desiccator (25 cm in diameter) containing ether until unconscious, removed to a clean cage until regaining consciousness, and then returned to the housing room. Animals were left to recover for 1 week before behavioral testing.

### Pre-SPS behavioral assay

#### Light-dark box

The light-dark box apparatus is a custom-built plastic box (42 × 42 cm) divided into two compartments: an open compartment with clear walls illuminated by the overhead lights in the testing room, and a dark compartment with opaque black walls and no interior illumination beyond what light enters through the entrance into the dark side of the box, thus simulating a secure, enclosed space. The compartments were connected by an opening at floor level. Each rat was gently placed in the lit compartment and allowed to explore freely for 6 min. A full crossing was defined as when the body of the rat was no longer visible from a top-down view of the box. Ethovision XT (Version 13) was used to assess time spent in the light and dark compartments, latency to cross into the dark compartment, and velocity.

### Post-SPS behavioral assays

#### Elevated plus maze

The EPM apparatus consists of a 10 cm^2^ center area, two open arms (50 × 10 cm), and two enclosed arms (50 × 10 × 30 cm). Rats were placed in one of the open arms, facing the center, and allowed to freely explore the apparatus for 5 min. Ethovision was used to assess time spent in the open and closed arms.

#### Open field and novel object recognition

Open field and novel object recognition (NOR) testing consisted of four phases performed over 2 days: Open Field (Day 1), Familiarization 1 (Day 1), Familiarization 2 (Day 2), and Novel Object Recognition Testing (Day 2). On day 1, rats were placed in the center of the empty box and allowed to freely explore for 10 min (Open Field). Approximately 2 h later, two objects (500 mL bottles filled with blue dye) were placed along the center axis of the box, approximately 1/3 distance from opposite walls, and rats were allowed to freely explore the objects for 10 min (Familiarization 1). On day 2, rats were placed back in with the same 2 objects from day 1 for 10 min (Familiarization 2). Four hours later, one of the objects was changed to a 12 × 12 × 7 cm tube holder, and rats were allowed to freely explore both objects for 10 min (Novel Object Recognition Testing). Novel and familiar object locations were counterbalanced within each group. Ethovision was used to assess center duration, border duration, and velocity for the open field phase. For the novel object recognition testing phase, Ethovision was used to assess time spent exploring (nose within 2 cm) the novel and familiar objects, as well as the velocity. Additionally, a novel object discrimination ratio was calculated using the following formula: (novel object duration − familiar object duration)/(novel object duration + familiar object duration).

#### Social interaction

Rats were placed in a clean cage (40 × 20 cm) with a novel age- and sex-matched conspecific for 10 min. Videos of the interactions were scored using Boris software by a blinded observer for total time conducting social (e.g., touching, sniffing), non-social (e.g., exploring the cage), aggressive (e.g., pinning), and submissive (e.g., presenting underside) behaviors of the experimental animal.

#### Auditory-cued fear conditioning

Fear conditioning was conducted in standard fear conditioning chambers (Lafayette Instrument Company, Lafayette, IN) housed within sound-attenuating cabinets and equipped with a stainless-steel grid floor capable of delivering footshock. All phases of the experiment (conditioning, extinction, and reinstatement) were conducted in the same context. Behavior was recorded using overhead cameras, and freezing behavior was quantified using Ethovision.

##### Acquisition

On Day 1, rats were placed in the chamber and allowed a 3-min baseline exploration period. Animals then received 6 pairings of an auditory conditioned stimulus (CS; 20-s tone, 4–8 kHz, ∼75–80 dB) co-terminating with an unconditioned stimulus (US; 0.42 mA footshock, 0.5 s duration). Intertrial intervals (ITIs) were 120 s. Rats remained in the chamber for 2 min following the final CS–US pairing before being returned to their home cages.

##### Extinction

Beginning 24 h after acquisition, rats underwent three consecutive days of extinction training (Days 2–4). Each extinction session began with a 2-min baseline period, followed by 7 CS-alone presentations (20-s tone, no shock) separated by 120-s ITIs. Freezing during CS presentations was measured to assess extinction learning across days.

##### Reinstatement test

Twenty-four hours after the final extinction session (Day 5), reinstatement testing was conducted. Rats were placed in the chamber and allowed a 2-min baseline period, after which 1 unsignaled footshock (0.42 mA, 0.5 s) was delivered to induce reinstatement. Following a 2 min post-shock interval, rats were presented with 5 CS-alone trials (20-s tone, no shock) separated by 120-s ITIs. Freezing during baseline and CS presentations following shock exposure was quantified as a measure of reinstated fear responding.

##### Behavioral scoring

Freezing, defined as the absence of all movement except that required for respiration, served as the primary index of conditioned fear. Percent time freezing during baseline and CS periods was calculated for conditioning, extinction, and reinstatement sessions.

### Immunohistochemistry

Ninety minutes post-fear conditioning reinstatement rats were administered an overdose of sodium pentobarbital and transcardially perfused with 0.1 M phosphate buffer (PBS) followed by 4% paraformaldehyde in 0.1 M PBS, pH 7.4. Brains were post-fixed in 4% paraformaldehyde at 4 °C for 24 h, then transferred to 30% sucrose in 0.1 M PBS at 4 °C until the moment of tissue processing. Brains were sliced into serial 35 μm coronal sections using a freezing microtome (−20 °C). Sections (1/6) were collected into wells containing cryoprotectant solution (30% Sucrose, 1% polyvinylpyrrolidone (PVP-40), and 30% Ethylene glycol, in 0.1 M PBS).

Two wells of tissue per animal were processed for c-Fos immunohistochemistry. Free-floating sections were rinsed in 0.1% phosphate-buffered saline (PBS) followed by an incubation in 1% sodium borohydride and then 1% hydrogen peroxide. Sections were blocked in 4% normal goat serum (NGS) containing 0.2% bovine serum albumin (BSA) and 0.2% Triton X-100.

Sections were incubated overnight at room temperature with c-Fos rabbit monoclonal primary antibody (1:500, Cell Signaling) diluted in blocking solution. The following day, sections were incubated with biotinylated goat anti-rabbit IgG secondary antibody (1:1,000, Vector Lab), followed by avidin-biotin peroxidase complex (ABC; Vectastain). Sections were stained using aqueous nickel sulfate enhanced 3,3′ diaminobenzidine tetrahydrochloride (DAB; Sigma). Sections were mounted from 0.3% aqueous gelatin, dehydrated, and cover-slipped with DPX.

Slide sections were scanned using a ZEISS axioscanner on the brightfield 10× setting. After scanning, images were uploaded to QuPath for analysis. QuPath is a open source bioimaging software that allows researchers to automate pathological analysis using a variety of tools. Cell counts were performed using QuPath’s positive cell detection tool. Each section was assessed for regions of interest (ROI) which were subsequently outlined and labeled under blinded conditions. The following regions were identified as ROIs where cell counts should be performed: Prelimbic area (PL), Infralimbic area (IL), paraventricular region (PVN), lateral amygdala (LA), basomedial amygdala (BMA), and the central nucleus of the amygdala (CEA). After going through all brain sections and outling ROIs a script was created to automate cell counts within these regions. The following settings were chosen as the best fit for this analysis. For setup, Hematoxylin OD was selected as the image type with a pixel size of 0.25 μm. Nucleus detection parameters were set to count cells with a minimum area of 15 μm^2^ and maximum area of 400 μm^2^. A threshold of 0.06 with a max background intensity of 2 was selected for intensity parameters.

### Statistics

Normality and homogeneity of variance were evaluated and if the assumption was violated, data were log transformed. Two-way ANOVA (stress × CORT-response), two-way repeated measures ANOVA (group × time), or repeated measure mixed-effects analysis (group × time) were used to assess behavioral endpoints within sex with a level of significance of *p* < 0.05 (GraphPad Prism 10.6.1). All *post hoc* testing utilized either Tukey’s or Holm Sidak multiple comparison tests, which were selected *a priori*. Pearson correlations tested associations among FC freezing to tone endpoints and AM, PM CORT determinations, CORT AUC following LD box exposure, and 1 day and 7 days post SPS CORT determinations. As no correlations survived FDR correction, we performed a permutation analysis to assess whether the observed directionality of relationships among variables was significant. The permutation test was conducted using the permuco package in R (Cinar and Viechtbauer, 2022; Frossard and Renaud, 2022), with secondary analysis using the poolr package, Detailed statistical results are provided in Supplementary Table 1.

## Results

### CORT response to mild and traumatic stress

To assess the role of CORT in predicting post-SPS behavior, CORT was measured multiple times throughout this study. First, pre-stress basal AM and PM CORT were measured. In both males and females, there were no significant differences in basal AM CORT measurements (Figures 2A, C), indicating any changes post-SPS are likely due to the stress itself, rather than a baseline difference among the groups. In females, there were also no differences in basal PM CORT (Figure 2D). In males, however, there was a significant interaction effect in basal PM CORT (interaction effect: *F*(1,16) = 7.508, *p* = 0.0145) and a main effect of CORT response (main effect of CORT response: *F*(1,16) = 9.835, *p* = 0.0064) (Figure 2B). To determine the high and low CORT responders, the area under the curve (AUC) was calculated from CORT measurements taken at 15, 30, 60, and 120 min post light-dark box exposure, with the upper quartile of the AUC for SPS and CTL groups making up the high CORT responders and the lower quartile making up the low CORT responders. In males, there was a significant interaction between time of CORT measurement and group, as well as main effects of both time and group on CORT concentrations post LD box exposure (see Supplementary Table 1 for statistics, Figure 3A). Females showed a main effect of both time and group on CORT concentration at 60 min post-LD box exposure (see Supplementary Table 1 for statistics, Figure 3C).

**FIGURE 2:**
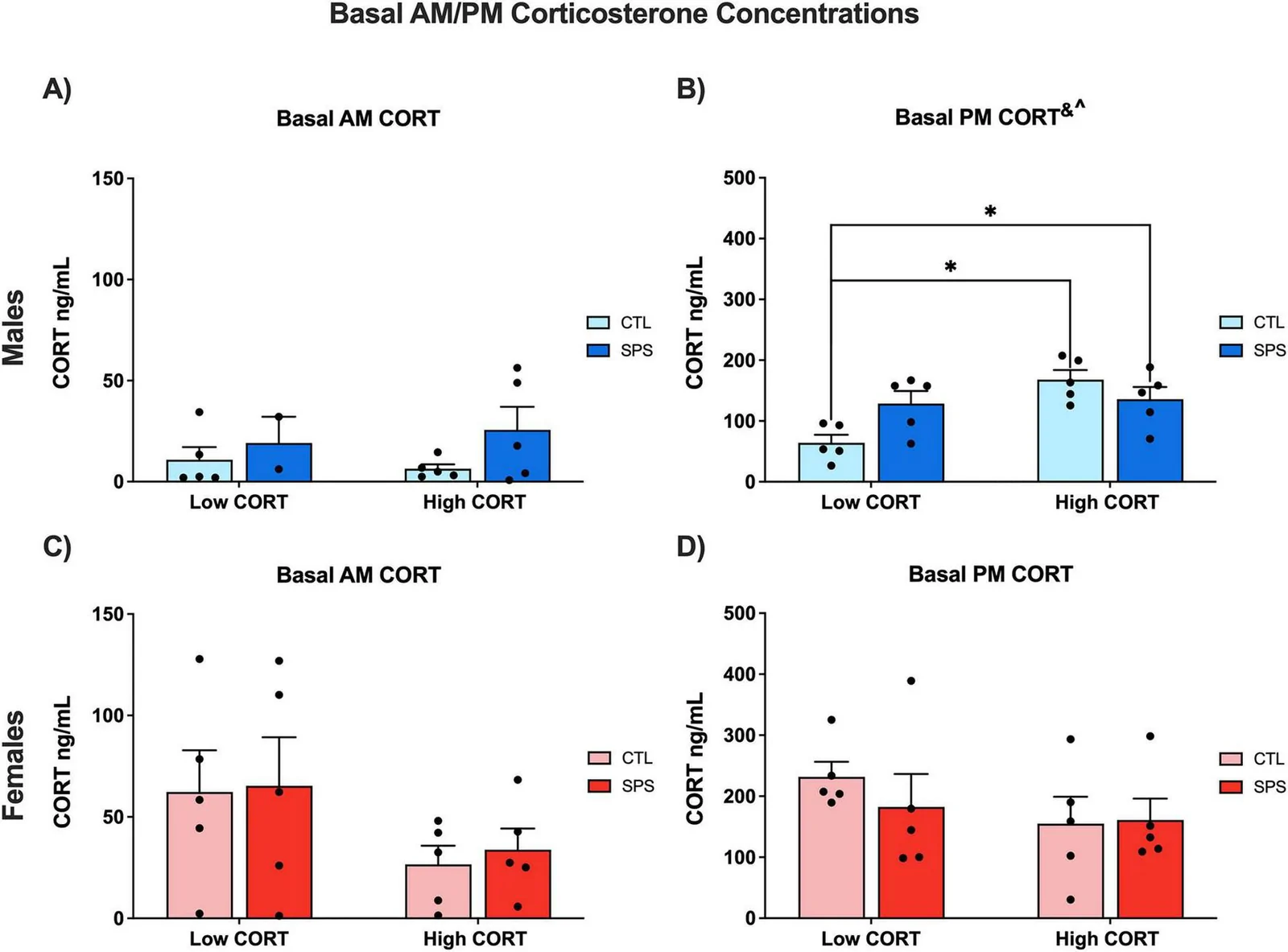
Effects of SPS and CORT response on basal AM and PM CORT concentrations. No significant difference in panel **(A)** basal AM CORT concentrations in males. Significant interaction between stress and CORT response and main effect of CORT response in panel **(B)** basal PM CORT concentrations in males. No significant difference in panel **(C)** basal AM or **(D)** PM CORT concentrations in females. All data were analyzed with two-way ANOVA. Mean ± SEM; *n* = 2–5/group; &, significant interaction; ^∧^, main effect of CORT; *post-hoc p ≤ 0.05.

**FIGURE 3:**
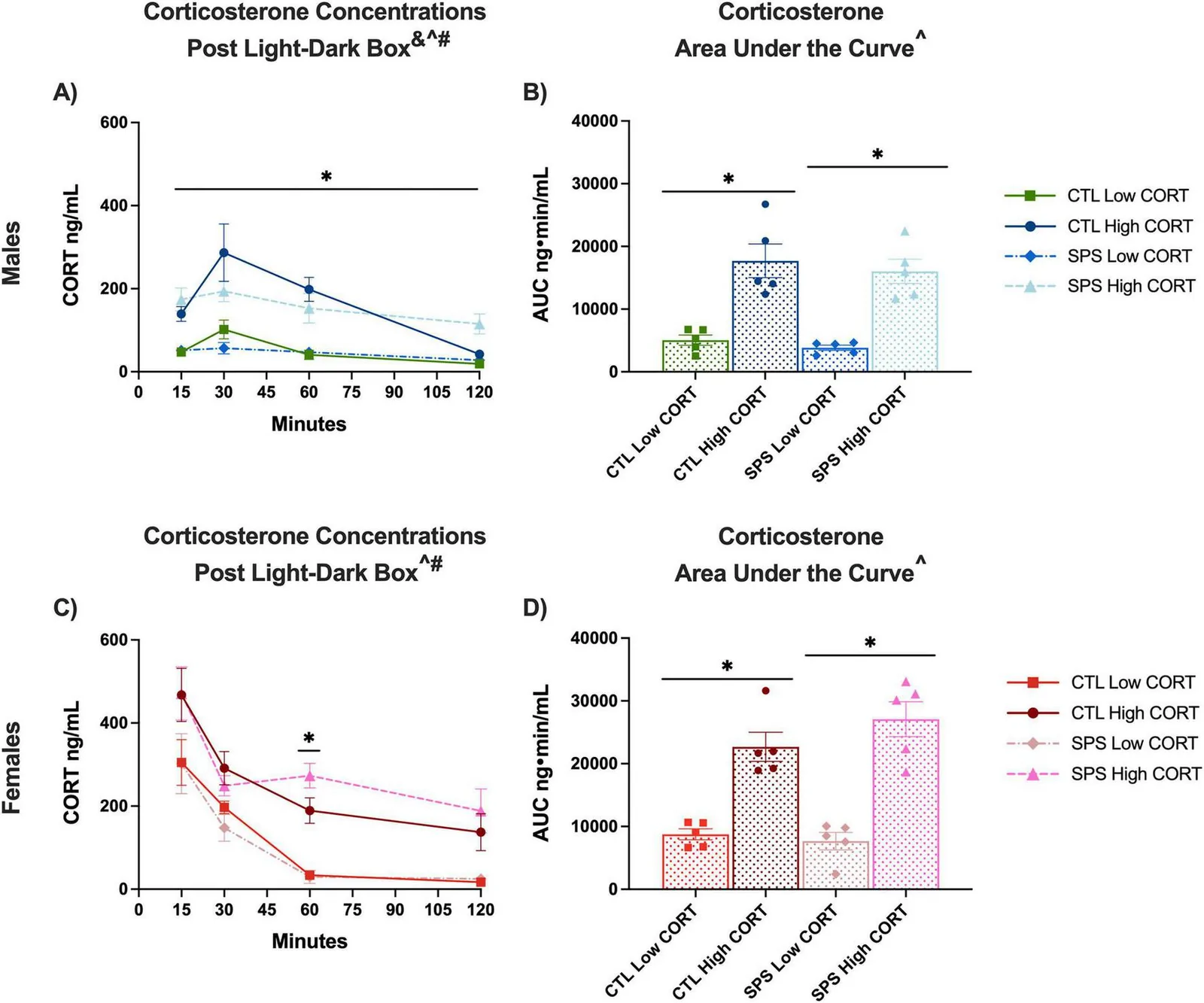
Effects of SPS and CORT response on area under the curve. Significant interaction between time and group and main effects of both time and group on panel **(A)** CORT concentrations post-LD box exposure in males. Main effect of CORT response on panel **(B)** area under the curve post-LD box exposure in males. Main effect of both time and group on panel **(C)** CORT concentration at 60 min post-LD box exposure in females. Main effect of CORT response on panel **(D)** area under the curve post-LD box exposure in females. Data were analyzed with either two-way ANOVA, repeated measures two-way ANOVA, or mixed-effects analysis. Mean ± SEM; *n* = 2–5/group; &, significant interaction; ^∧^, main effect of time **(A,C)**; ^∧^, main effect of CORT **(B,D)**; #, main effect of group **(A,C)**; #, main effect of stress **(B,D)**; *post-hoc p ≤ 0.05.

As expected, the AUC was affected by CORT responder status in both males and females (main effect of CORT: see Supplementary Table 1 for statistics, Figures 3B, D). In addition to these CORT measurements, we should note that there were no significant differences among groups in light side and dark side duration, latency to enter the dark side of the box, and velocity during the light-dark box task in both males and females (Supplementary Figure 1). This suggests that the relationship between mild stress-induced CORT levels and post-SPS behavior is reflected by individual variation in mild physiological stress responsivity rather than baseline behavioral differences. Basal AM CORT measurements were also taken 1 day and 7 days post-SPS exposure. While males did not exhibit any changes in basal AM CORT 1 day or 7 days post-SPS exposure (Figures 4A, B), females showed a main effect of stress both 1 (*F*(1,16) = 6.193, *p* = 0.0242; Figure 4C) and 7 days (*F*(1,16) = 7.726, *p* = 0.0134; Figure 4D) post-SPS, with SPS-exposed females having significantly lower basal AM CORT measurements than their control counterparts. Additional statistical analyses examining basal AM CORT values within group across pre-SPS, 1 day, and 7 days post-SPS revealed a main effect of day in both males (*F*(1.307, 18.94) = 11.04, *p* = 0.002; Supplementary Figure 2A) and females (*F*(1.415, 22.64) = 29.84, *p* < 0.0001; Supplementary Figure 2B).

**FIGURE 4:**
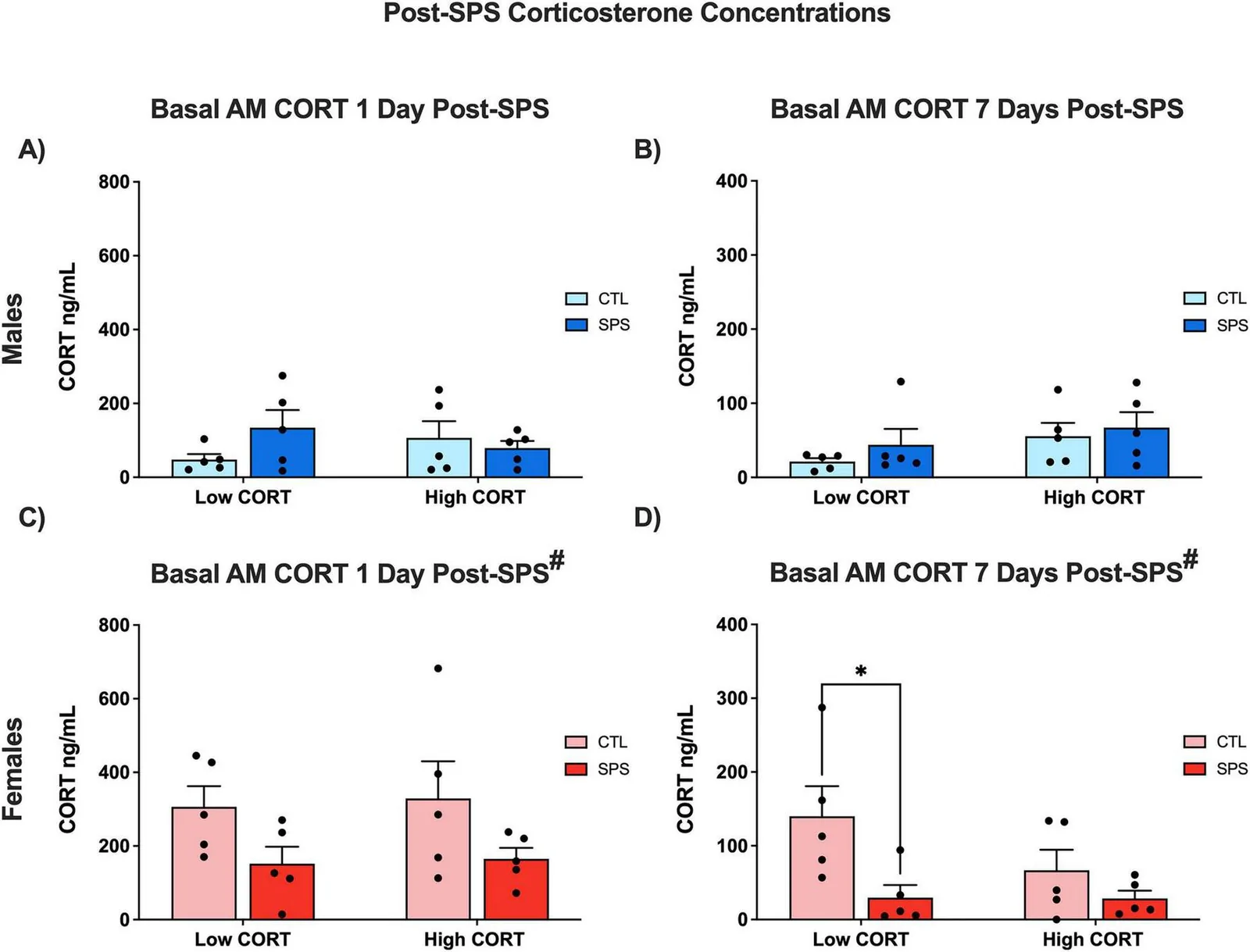
Effects of SPS and CORT response on basal AM CORT concentrations 1 and 7 days post-SPS. No significant difference in basal AM CORT concentrations **(A)** 1 day and **(B)** 7 days post-SPS in males. Main effect of stress on basal AM CORT concentration **(C)** 1 day and **(D)** 7 days post-SPS in females. All data were analyzed with two-way ANOVA. Mean ± SEM; *n* = 5/group; #, main effect of stress; *post-hoc p ≤ 0.05.

### Impact of SPS and CORT response to a mild stressor on behavior

In males, neither SPS nor CORT response to mild stress affected the time spent (duration) in the open and closed arms of the EPM (Figures 5A, B). However, females exposed to SPS as well as female high CORT responders displayed decreased duration in the open arms (main effect of stress: *F*(1,16) = 9.558, *p* = 0.007; main effect of CORT response: *F*(1,16) = 4.979, *p* = 0.0403; Figure 5C) and increased duration in the closed arms (main effect of stress: *F*(1,16) = 14.07, *p* = 0.0017; main effect of CORT response: *F*(1,16) = 9.065, *p* = 0.0083; Figure 5D) of the EPM. There were no effects of SPS or CORT response in center and border duration during the open field task in either sex (Supplementary Figure 3). During novel object recognition, no differences in familiar and novel object investigation duration or velocity were observed due to SPS or CORT response in males or females (Supplementary Figure 4). Additionally, the discrimination ratio was not altered in males or females (Supplementary Figure 5). The social interaction test did not reveal alterations in aggressive behavior duration, submissive behavior duration, rearing duration (Supplementary Figure 6), or total duration of social behaviors in males or females (Figures 6A, C). While females did not exhibit any changes in self-grooming during the social interaction test (Figure 6D), SPS significantly decreased self-grooming duration during the social interaction procedure in males (main effect of stress: *F*(1,16) = 12.06, *p* = 0.0031; Figure 6B).

**FIGURE 5:**
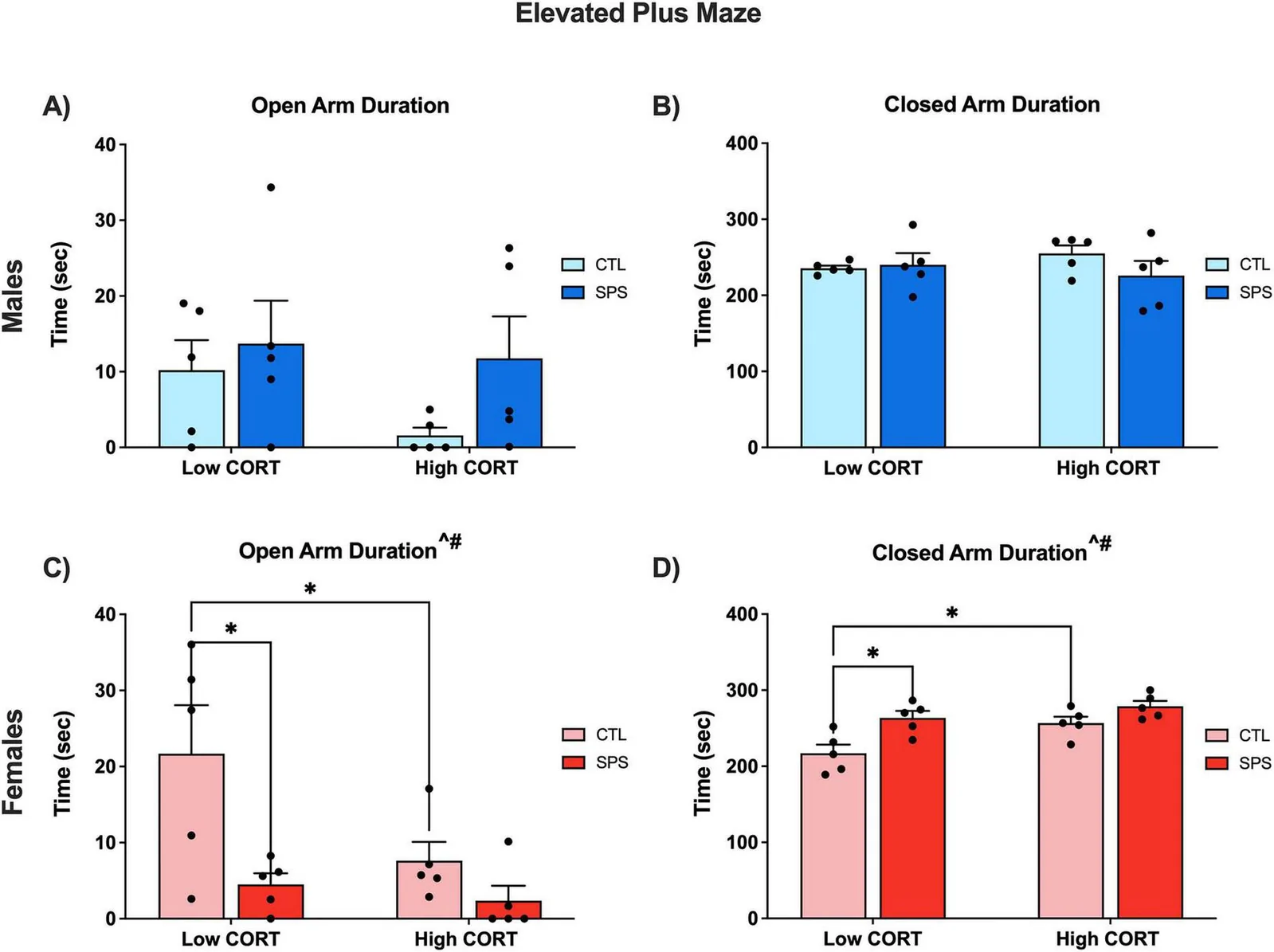
Effects of SPS and CORT response on elevated plus maze. No significant difference in time spent (duration) in the **(A)** open or **(B)** closed arms of the EPM in males. Main effect of stress and CORT response on time spent (duration) in the **(C)** open and **(D)** closed arms of the EPM in females. All data were analyzed with two-way ANOVA. Mean ± SEM; *n* = 5/group; ^∧^, main effect of CORT; #, main effect of stress; *post-hoc p ≤ 0.05.

**FIGURE 6:**
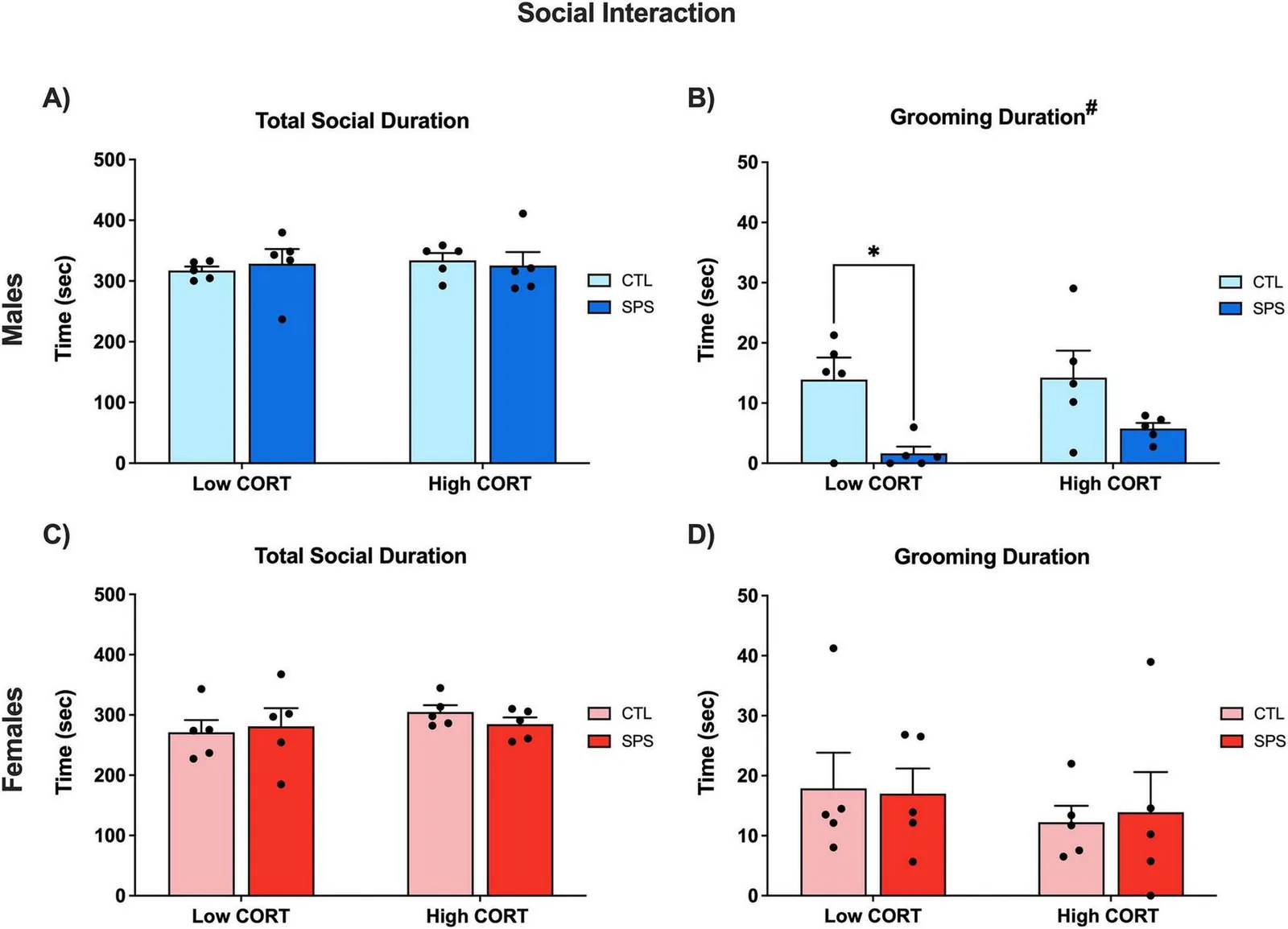
Effects of SPS and CORT response on social interaction. No significant difference in the **(A)** total duration of social behaviors performed in males. Main effect of stress on panel **(B)** grooming duration in males. No significant difference in the **(C)** total duration of social behaviors performed or **(D)** grooming duration in females. All data were analyzed with two-way ANOVA. Mean ± SEM; *n* = 5/group; #, main effect of stress; *post-hoc p ≤ 0.05.

In addition to the aforementioned assays, the rats also performed auditory-cued fear conditioning. All groups rapidly and effectively learned to freeze to the CS–US pairings across acquisition trials (Figures 7A, D). All groups showed extinction of conditioned fear across the 3 days of extinction trial exposure. Males displayed increased freezing in the control high CORT responders across all three extinction days (main effect of extinction day: *F*(2, 48) = 11.90, *p* < 0.0001; main effect of group: *F*(3, 48) = 3.886, *p* = 0.0145; Figure 7B) while females displayed increased freezing among the SPS-exposed high CORT responders across all three extinction days (main effect of extinction day: *F*(2, 48) = 10.87, *p* = 0.0001; main effect of group: *F*(3, 48) = 4.472, *p* = 0.0076; Figure 7E). A significant interaction effect was observed during reinstatement tones in females (interaction effect: *F*(1,16) = 5.031, *p* = 0.0394; Figure 7F), indicating that high-CORT responder females exposed to SPS froze more during the reinstatement tone presentation phase of auditory-cued fear conditioning. In contrast, there was no effect of SPS or mild stress reactivity on reinstatement in males (no change in freezing duration during reinstatement tone presentation) (Figure 7C).

**FIGURE 7:**
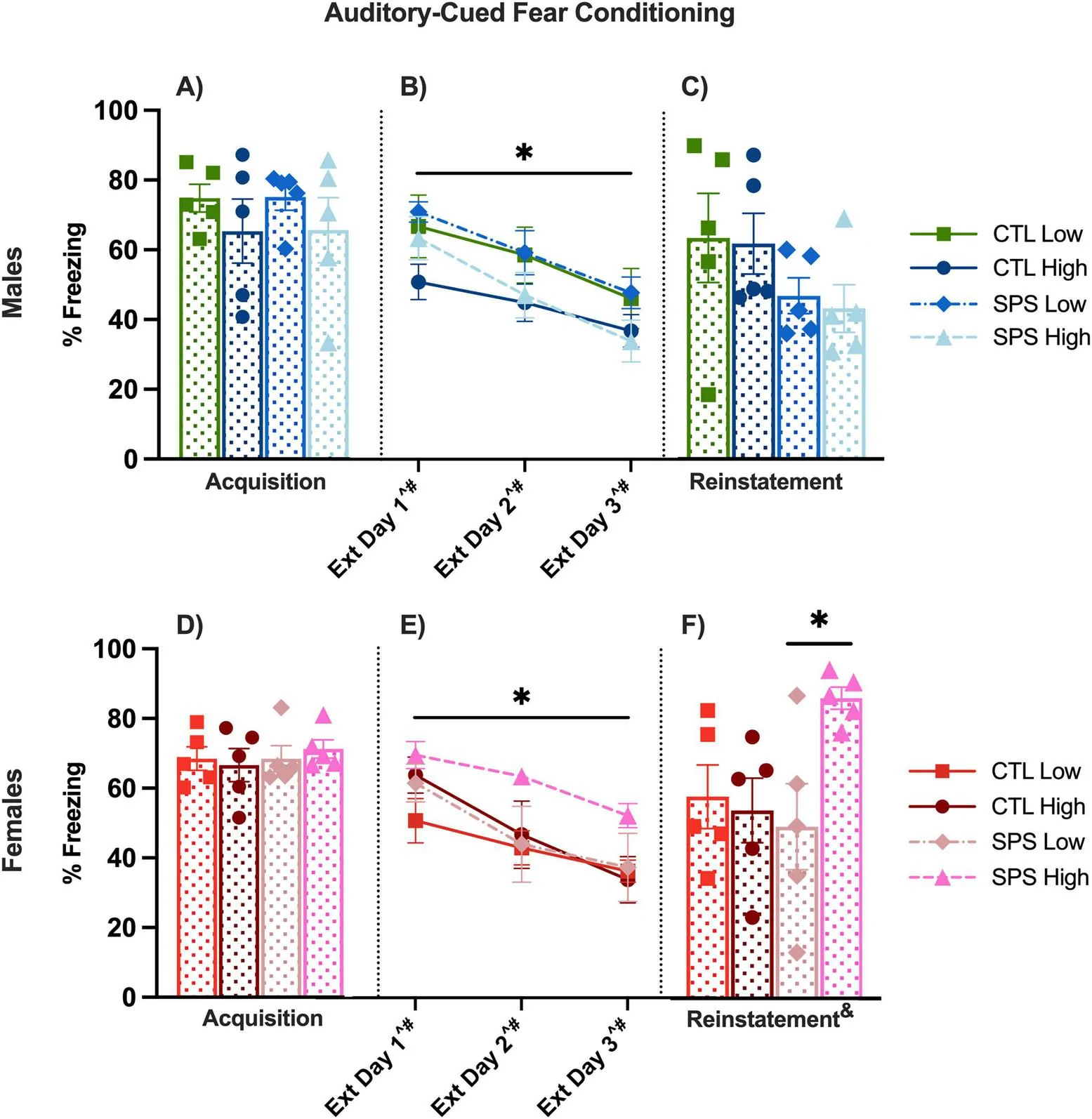
Effects of SPS and CORT response on auditory-cued fear conditioning. No significant difference in percent of time spent freezing during **(A)** acquisition or **(C)** reinstatement tones in males. Main effect of extinction day and group on percent of time spent freezing during **(B)** extinction tones across days 1–3 in males. No significant difference in percent of time spent freezing during **(D)** acquisition tones in females. Main effect of extinction day and group on percent of time spent freezing during **(E)** extinction tones across days 1–3 in females. **(F)** Significant interaction between stress and CORT response on percent of time spent freezing during reinstatement tones in females. All data were analyzed with two-way ANOVA. Mean ± SEM; *n* = 5/group; &, significant interaction; ^∧^, main effect of day; #, main effect of group; *post-hoc p ≤ 0.05.

Exploratory Pearson correlations were utilized to explore relationships among fear conditioning variables and CORT measurements. In SPS-exposed females, CORT AUC showed nominally significant positive associations with reinstatement freezing, whereas negative associations with pre-stress AM CORT were observed during extinction and reinstatement endpoints (Figure 8, nominal *p*’s < 0.05). None survived Benjamini-Hochberg correction (*q* > 0.18), likely due to the number of variables tested and the low sample size for this type of analysis. We then performed permutation analyses to assess directional effects across the dataset. Permutation analyses confirmed directional consistency across freezing outcomes in the female SPS group, with lower pre-stress AM CORT predicting increased freezing (negative direction, all three permutation methods, *p* ≤ 0.05) and higher mild stress-induced CORT AUC predicting increased freezing (positive direction, permuco *p* = 0.001). This disassociation between basal and mild stress-induced directional relationships was unique to females and supports a relationship between CORT dynamics and behavior.

**FIGURE 8:**
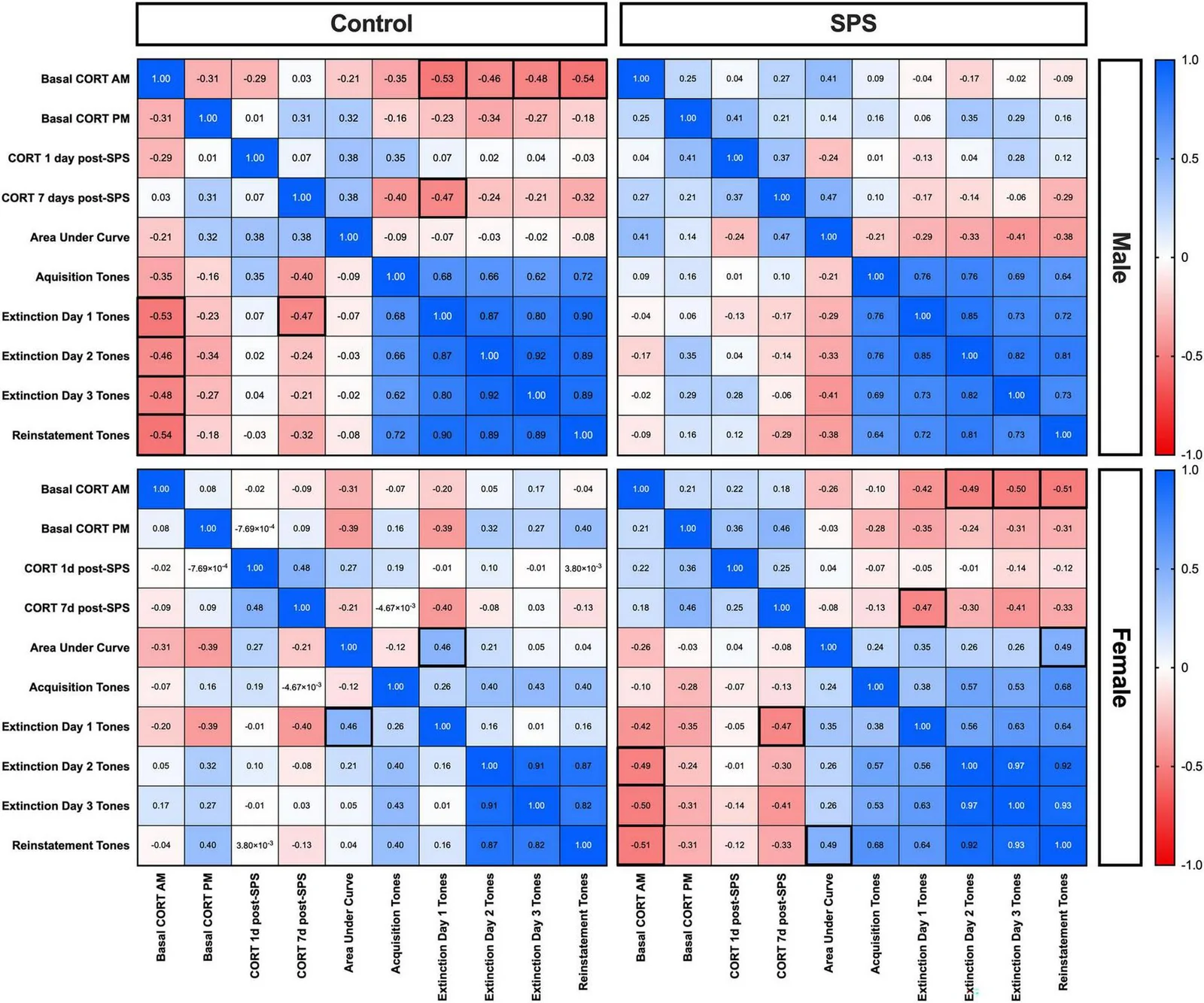
Fear conditioning freezing behavior and CORT response correlation matrix. **(**Upper Left**)** control males, **(**Upper Right**)** SPS males, **(**Lower Left**)** control females, **(**Lower Right**)** SPS females. All data were analyzed with Pearson’s Correlation. Mean ± SEM, *n* = 20/group, numbers within boxes = *r*-values, bolded box = nominal *p* ≤ 0.05.

While CORT AUC showed a nominally significant positive association with extinction day 1 freezing in control females (Figure 8, nominal *p*’s < 0.05), this finding did not survive FDR correction, and permutation analyses did not reveal a significant directional effect between the two factors. As with the females, males exhibited a negative association between pre-stress AM CORT and extinction/reinstatement freezing behavior (lower AM CORT predicts greater freezing). However, this relationship emerged in the control male group (again with no individual values surviving FDR correction), whereas it emerged in the SPS-exposed female group (Figure 8, nominal *p*’s < 0.05). Additionally, permutation analyses indicated a significant directional effect of pre-stress AM CORT on freezing behavior in males. In all, these data suggest sex-specific differences in the potential predictive value of CORT dynamics on traumatic stress-induced behavior.

### Impact of SPS and CORT response on cFos expression across brain regions

Animals were euthanized, and brains were perfused 90 min following the reinstatement phase of fear conditioning to explore the effects of SPS and CORT responder status on stress-induced neuronal activation across brain regions. cFos, a molecular correlate of neuronal activity, was measured via DAB staining in the prefrontal cortex (PL), infralimbic cortex (IL), paraventricular nucleus of the hypothalamus (PVN), lateral amygdala (LA), basomedial amygdala (BMA), and the central amygdala (CEA) of the male and female rats. Neither SPS nor CORT responder status affected the average number of cFos positive neurons per mm^2^ in the prelimbic cortex in males (Figure 9A) or females (Figure 10A). In the IL, control high CORT responder males showed increased average number of cFos positive cells per mm^2^ (main effect of CORT: *F*(1, 15) = 7.778, *p* = 0.0138; Figure 9B), while there were no IL differences in females (Figure 10B). In the PVN, SPS-exposed high CORT responder females displayed an elevated average number of cFos positive cells per mm^2^ (interaction effect: *F*(1, 16) = 7.566, *p* = 0.0142; Figure 10C), indicating increased neuronal activity in the PVN of SPS-exposed, high CORT responder females. Increased neuronal activity in the PVN was also observed among males, with control high CORT responders showing an elevated average number of cFos positive cells per mm^2^ (interaction effect: *F*(1, 15) = 6.253, *p* = 0.0245; main effect of CORT: *F*(1, 15) = 9.182, *p* = 0.0084; Figure 9C). While no differences in cFos expression were observed among males in the LA (Figure 9D), BMA (Figure 9E), or CEA (Figure 9F), we found decreased cFos expression among high CORT responder females in the LA (main effect of CORT: *F*(1, 16) = 7.501, *p* = 0.0146; Figure 10D), BMA (main effect of CORT: *F*(1, 15) = 5.232, *p* = 0.0371; Figure 10E), and CEA (main effect of CORT: *F*(1, 16) = 4.976, *p* = 0.0404; Figure 10F), indicating diminished neuronal activity among high CORT responder females in these brain regions post-reinstatement. Interestingly, we observed increased cFos expression among SPS-exposed females in the CEA as well, suggesting SPS increases neuronal activity among females in the CEA (main effect of stress: *F*(1, 16) = 4.793, *p* = 0.0437; Figure 10F).

**FIGURE 9:**
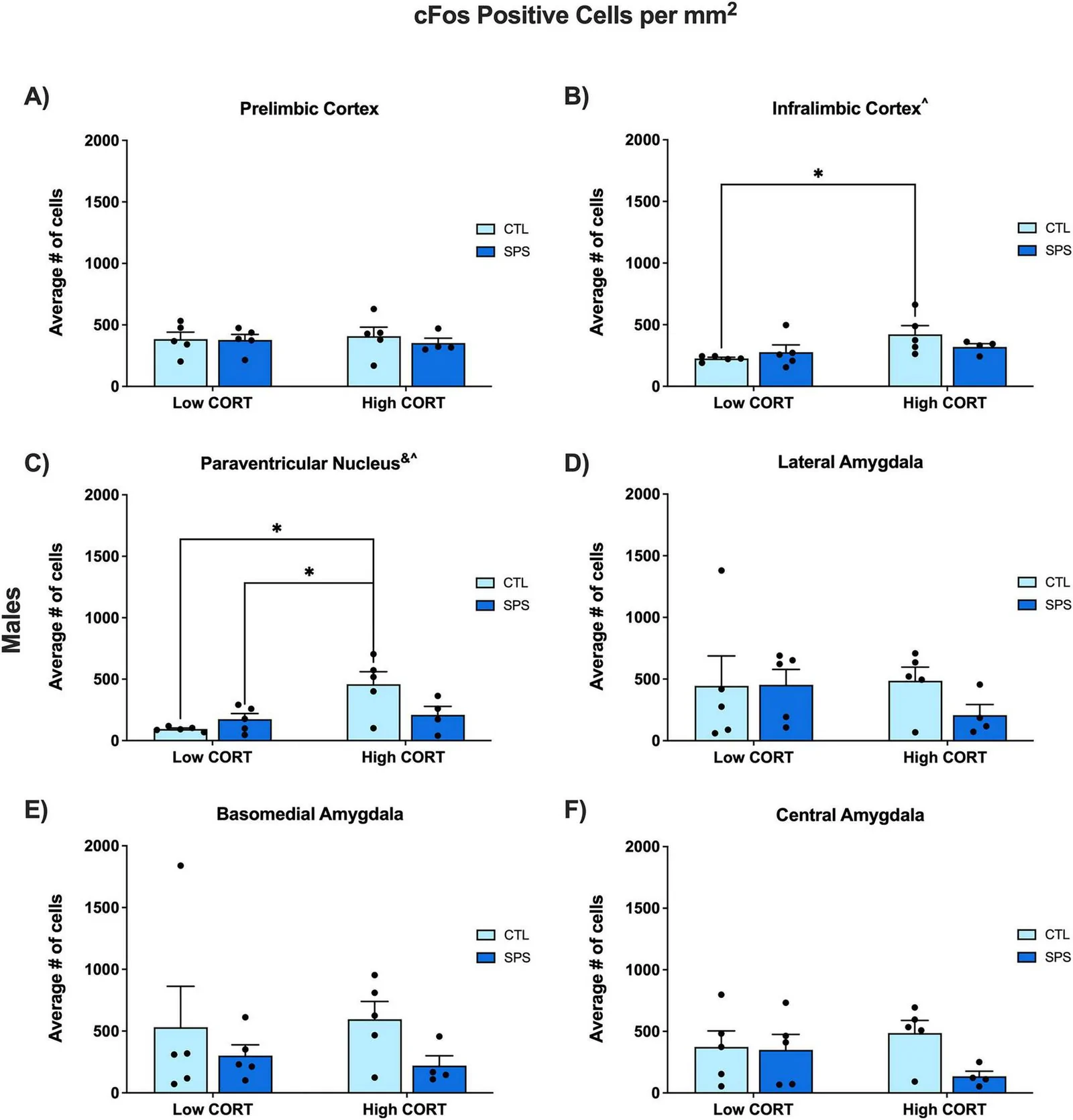
Effects of SPS and CORT response on cFos positive neurons per mm^2^ in males. No significant difference in the average number of cFos positive neurons per mm^2^ in the **(A)** prelimbic cortex, **(D)** lateral amygdala, **(E)** basomedial amygdala, or the **(F)** central amygdala in males. Main effect of CORT response in the average number of cFos positive neurons per mm^2^ in the **(B)** infralimbic cortex in males. Significant interaction between stress and CORT response and main effect of CORT response in the average number of cFos positive neurons per mm^2^ in the **(C)** paraventricular nucleus. All data were analyzed with two-way ANOVA. Mean ± SEM; *n* = 5/group; &, significant interaction; ^∧^, main effect of CORT; #, main effect of stress; *post-hoc p ≤ 0.05.

**FIGURE 10:**
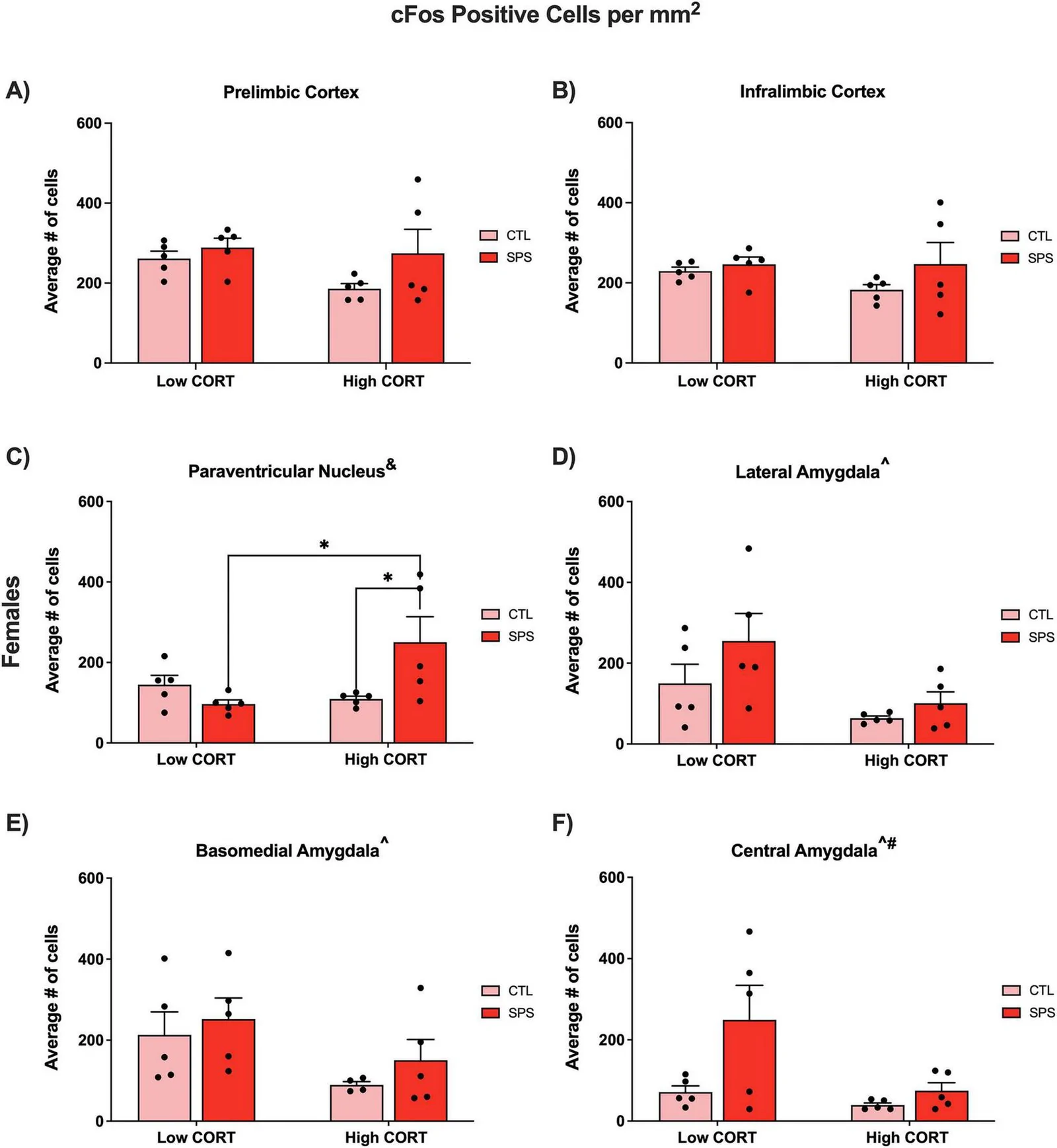
Effects of SPS and CORT response on cFos positive neurons per mm^2^ in females. No significant difference in the average number of cFos positive neurons per mm^2^ in the **(A)** prelimbic or **(B)** infralimbic cortex in females. Significant interaction between stress and CORT response in the average number of cFos positive neurons per mm^2^ in the **(C)** paraventricular nucleus. Main effect of CORT response in the average number of cFos positive neurons per mm^2^ in the **(D)** lateral amygdala and **(E)** basomedial amygdala. Main effect of CORT response and stress on the average number of cFos positive neurons per mm^2^ in the **(F)** central amygdala in females. All data were analyzed with two-way ANOVA. Mean ± SEM; *n* = 5/group; &, significant interaction; ^∧^, main effect of CORT; #, main effect of stress; *post-hoc p ≤ 0.05.

## Discussion

In the present study, we investigated whether HPA axis reactivity to a mild stressor (light-dark box) can predict behavioral outcomes following later exposure to traumatic stress (SPS). The results demonstrate that CORT responses (AUC) to a light-dark box distinguish high and low stress-responsive individuals and that these differences may be useful in predicting sex-dependent traumatic stress phenotypes. Specifically, in females, heightened stress reactivity to mild stress was associated with enhanced reinstatement of fear memory following SPS. In contrast, CORT measures do not reliably predict behavioral performance following SPS in males but may be a predictor of fear reactivity later in life. Together, these findings suggest a sex-specific relationship between mild stress reactivity and resting glucocorticoid dynamics with susceptibility or resilience to traumatic stress.

Previous research has demonstrated individual variability in stress reactivity across behavioral and neuroendocrine outcomes (Belda et al., 2020; Cohen et al., 2006; Daws et al., 2017; Galatzer-Levy et al., 2014; Skórzewska et al., 2020; Song et al., 2008; Steudte-Schmiedgen et al., 2015; Yehuda et al., 1998). This variability is thought to influence vulnerability to stress-related disorders such as PTSD by shaping how organisms respond to both acute and chronic stressors (Bomyea et al., 2012; Cohen et al., 2003; Danan et al., 2021; Daws et al., 2017; McEwen, 2004; Zaba et al., 2015). Our study suggests that CORT responses to a mild stressor, such as the light-dark box, may be used as a meaningful index of long-term reactivity to a pronounced stress exposure.

Single-prolonged stress exposure consistently results in significant alterations in post-stress CORT levels, with numerous studies indicating that SPS decreases post-stress plasma CORT levels and enhances dexamethasone suppression of CORT response (Daws et al., 2017; Kohda et al., 2007; Lisieski et al., 2018; Perrine et al., 2016; Yamamoto et al., 2009). It is important to note that the vast majority of these studies were performed only in male animals. We did not detect any significant changes in post-SPS plasma CORT levels among males. However, basal AM plasma CORT levels both 1 and 7 days post-SPS were significantly reduced in females, a pattern consistent with the hypocortisolism frequently observed in PTSD populations (Daskalakis et al., 2016; de Kloet et al., 2006; Galatzer-Levy et al., 2014; Steudte-Schmiedgen et al., 2015; von Majewski et al., 2023) as well as the aforementioned rodent studies. We were surprised to see elevated basal AM CORT levels in control females after SPS occurred (1 and 7-days post-SPS), even though they were not exposed to the SPS protocol. This increase may reflect sensitization from prior exposure to mild stress during the light-dark box assay, a pattern absent in the SPS-exposed group likely due to the CORT-blunting effects of SPS. Together, these findings indicate that SPS alters HPA axis dynamics, but that the nature and detectability of these changes differ between sexes.

We observed a robust PTSD-like phenotype in females, with mild stress reactivity predicting certain behavioral outcomes following SPS-exposure. High CORT responders exhibited increased anxiety-like behavior in the elevated plus maze, as evidenced by reduced open arm and increased closed arm duration. In addition, these animals exhibited increased freezing during extinction and reinstatement, indicating persistent fear responses. Correlational analyses revealed a positive association between post-mild stress CORT AUC and freezing duration during reinstatement, as well as negative associations between pre-stress AM CORT levels and freezing behavior during the extinction and reinstatement phases of auditory-cued fear conditioning. Permutation analysis confirmed a directional relationship between pre-stress AM CORT and CORT AUC on extinction and reinstatement freezing in SPS females. These results are consistent with prior work implicating HPA axis dysregulation in fear learning and extinction processes (Aubry et al., 2016; de Quervain et al., 2017; de Quervain et al., 2019; Jovanovic et al., 2010; Roozendaal et al., 2006). Elevated glucocorticoid responses during stress may enhance consolidation of aversive memories (Aubry et al., 2016; Cordero and Sandi, 1998; de Quervain et al., 2017; Roozendaal et al., 2006), while subsequent reductions in basal glucocorticoids may impair extinction and promote persistent fear expression (de Quervain et al., 2019; Gourley et al., 2009; Keller et al., 2015; Monari et al., 2024). Taken together, these findings indicate that low baseline basal CORT levels combined with heightened mild stress reactivity may promote maladaptive behavioral outcomes post-SPS in females and may be measures for predicting PTSD susceptibility.

In control males, reduced pre-stress CORT predicts increased fear expression (impaired fear extinction and enhanced reinstatement). Unlike females, there was no relationship evident between measures of CORT dynamics and freezing behavior following SPS. While SPS reduced self-grooming behavior during the social interaction behavioral assay, this effect was not modulated by CORT responder status. Overall, these results reveal that glucocorticoid responsivity may not be a sufficient predictor of behavioral stress susceptibility in males and highlight pronounced male-female differences in how glucocorticoid dynamics impact fear processing and reactivity to a traumatic stress.

We also found that SPS exposure and mild stress reactivity may interact to modulate neuronal activation within the paraventricular nucleus (PVN) following reinstatement, with SPS-exposed high CORT-responder females exhibiting increased cFos expression. Given that cFos reflects activity-dependent transcription, this finding suggests that prior mild stress responsivity could shape corticotropin-releasing hormone (CRH) neuron recruitment in the PVN following traumatic stress exposure. The PVN integrates limbic input and glucocorticoid feedback (Herman and Tasker, 2016; Herman et al., 2003; Ulrich-Lai and Herman, 2009; Zheng et al., 2026), and increased activation in high responders may reflect altered feedback sensitivity. The differential cFos activation in PVN occurs in the context of elevated freezing (but not HPA axis response) in the high CORT group, suggesting a possible relationship between neuronal and behavioral endpoints. In males, increased cFos-positive cell density in the control high CORT responders indicates that PVN activation is associated with CORT responder status rather than SPS exposure. Additionally, control high CORT responders exhibited increased cFos-positive cell density within the infralimbic cortex relative to control low responders. The infralimbic cortex plays an important role in fear extinction-related processes (Milad and Quirk, 2002; Quirk and Mueller, 2008), and increased activation in high responders may reflect trait-like differences in medial prefrontal engagement associated with stress reactivity. Notably, no SPS-dependent alterations in infralimbic activity were observed, and no amygdala differences emerged in males, further supporting the idea that mild stress reactivity has a less robust relationship with trauma-related phenotypes in males compared to females.

Conversely, females exhibited reduced reinstatement-induced cFos expression in the lateral and basomedial amygdala among high CORT responders, alongside decreased resting central amygdala activity overall but increased activation following SPS. These regions are critical for fear learning and expression (Janak and Tye, 2015; LeDoux, 2000; Roozendaal et al., 2009), and this activation pattern may reflect altered emotional processing and heightened fear output in high CORT responders following trauma (Phelps and LeDoux, 2005; Ressler and Mayberg, 2007). No differences were observed in prelimbic or infralimbic cortex, suggesting limited SPS-induced recruitment of medial prefrontal regulatory circuits during reinstatement. Additionally, the absence of amygdala effects in males further supports sex-specific patterns of circuit engagement underlying stress-related phenotypes.

Our observations are consistent with a large body of literature demonstrating sexually diergic regulation of stress systems. Consistent with our findings, females tend to exhibit greater HPA axis activation in response to stress (Goel et al., 2014; Handa et al., 2022; Heck and Handa, 2019), suggestive of enhanced stress reactivity and reduced negative feedback (Babb et al., 2013; Kolber and Muglia, 2009; Solomon et al., 2015). In rodents, female HPA axis hyperactivation is associated with disproportionately larger adrenal glands (Handa et al., 2022; Selye, 1937), while differences in glucocorticoid receptor expression, sensitivity, and feedback efficiency likely contribute to sex-specific HPA responses across rodents and humans (Handa et al., 2022; Heck and Handa, 2019; Kolber and Muglia, 2009; Solomon et al., 2015). These differences are further shaped by gonadal hormones, with estrogen amplifying adrenal responsivity and HPA reactivity in females, and androgens generally dampening HPA reactivity in males (Handa et al., 1994, 2022; Heck and Handa, 2019; Viau and Meaney, 1991; Viau and Meaney, 1996). Females also have significantly elevated levels of corticosteroid-binding globulin, which effectively reduces free corticosteroid levels relative to males (Minni et al., 2014; Stolk et al., 1996; Toews et al., 2023). At the circuit level, sexual differentiation in stress-related brain regions, such as the amygdala, hippocampus, and prefrontal cortex, may contribute to the observed divergent behavioral outcomes (Farrell et al., 2013; Premachandran et al., 2020). These regions are critical for fear learning, extinction, and contextual processing, and can exhibit sex-dependent plasticity in response to stress (Graham et al., 2026; Lebron-Milad and Milad, 2012; McEwen et al., 2016; Milad and Quirk, 2012). Taken together, these sex-specific differences may contribute to the increased prevalence of PTSD in women (Koenen et al., 2017) and support our finding that mild stress reactivity may be a useful predictor of PTSD susceptibility in females.

While utilizing a quartile split is a commonly accepted way to stratify extreme populations (Preacher et al., 2005; Yong et al., 2016), doing so reduces the sample size of each group, which limits statistical power. It is also important to note that although our study shows elevated mild stress reactivity aligns with traumatic stress-related pathologies in females, these findings are largely correlational, and further studies manipulating glucocorticoid signaling are necessary before making more definitive conclusions regarding whether elevated mild stress reactivity directly contributes to susceptibility or simply serves as an associated marker. To be conservative, we ran males and females in separate cohorts at different times. As a result, we can only observe differences in stress reactivity within each sex, and future studies should be designed so that true sex differences can be examined. Lastly, estrus cycle stage is thought to affect the female HPA-axis response, as depicted by differences in plasma corticosterone levels (Handa et al., 2022; Viau and Meaney, 1991). To minimize extraneous disturbances across behavioral testing, we did not assess the effects of estrus cycle stage on stress reactivity, which may be an important factor to consider in future studies.

In summary, the present study demonstrates that mild stress reactivity can predict susceptibility to SPS-induced behavioral and endocrine dysregulation in females, but not in males. These results highlight the importance of incorporating sex as a biological variable in preclinical models of stress and highlight the importance of sex-specific mechanisms in shaping trauma-related outcomes. In all, our findings help advance our knowledge of stress pathophysiology and identify variables that may contribute to PTSD susceptibility, suggesting the potential to better identify and treat at-risk individuals. Future studies should investigate the neural and molecular mechanisms underlying these effects, including glucocorticoid receptor signaling and circuit-level changes in stress-related brain regions. Additionally, larger cohort studies including the investigation of hormone status will be critical for validating these findings and advancing our understanding of individual differences in stress susceptibility.

## Data Availability

The original contributions presented in this study are included in this article/Supplementary material, further inquiries can be directed to the corresponding authors.
