# Supplementary material for: Glucocorticoid dynamics predict susceptibility and resilience to traumatic stress in male and female rats
Source: Front Cell Neurosci. 2026 Oct 9;20:1880559. doi: 10.3389/fncel.2026.1880559 (PMC13691938; doi:10.3389/fncel.2026.1880559)
Supplement: Supplementary file 1 [file Supplementary_file_1.docx]

-SUPPLEMENTARY INFORMATION FOR-

**Glucocorticoid dynamics predict susceptibility and resilience to traumatic stress in male and female rats**

**Devine EA^1,2^, Chambers J^1^, Dunn T^1^, Musarra A^1^, Porter S^1,2^, Moore C^1,2^, Packard B^1^, Herman JP^1,3*^**

^1^Department of Pharmacology, Physiology, and Neurobiology, University of Cincinnati, Cincinnati, OH, USA

^2^Neuroscience Graduate Program, University of Cincinnati, Cincinnati, OH, USA

^3^Veterans Affairs Medical Center, Cincinnati, OH, USA

**Table of Contents**

**Supplementary Figures**

- **Supplementary Figure 1**: Light-Dark Box Behavioral Results
- **Supplementary Figure 2**: Pre- & Post-SPS Basal AM Corticosterone Concentrations
- **Supplementary Figure 3**: Open Field Behavioral Results
- **Supplementary Figure 4**: Novel Object Recognition Behavioral Results
- **Supplementary Figure 5**: Novel Object Recognition Discrimination Ratio Results
- **Supplementary Figure 6**: Additional Social Interaction Behavioral Results

**Supplementary Table** (as *separate Excel file*):

- **Supplementary Table 1**: Detailed Statistical Results

**SUPPLEMENTARY FIGURES**

**Supplementary Figure 1. Effects of SPS and CORT Response on Light-Dark Box.** No significant difference in **A)** light side and **B)** dark side duration in males. No significant difference in **C)** latency to cross into the dark side of the box and no significant difference in **D)** velocity in males. No significant difference in **E)** light side and **F)** dark side duration in females. No significant difference in **G)** latency to cross into the dark side of the box and no significant difference in **H)** velocity in females. All data were analyzed with two-way ANOVA. Mean+/- SEM, n=5/group.

**Supplementary Figure 2. Effects of Day and Group on Pre- and Post-SPS Basal AM CORT Concentrations.** Main effect of day on basal AM CORT concentrations in **A)** males and **B)** females. All data were analyzed with either two-way repeated measures ANOVA (time/day x group), or mixed-effects analysis. Mean+/- SEM, n=2-5/group, ^ = Main effect of day, *post-hoc p $\leq$0.05.

**Supplementary Figure 3. Effects of SPS and CORT Response on Open Field.** No significant difference in time spent (duration) in the **A)** center or **B)** border of testing arena in males. No significant difference in time spent (duration) in the **C)** center or **D)** border of testing arena in females. All data were analyzed with two-way ANOVA. Mean+/- SEM, n=5/group.

**Supplementary Figure 4. Effects of SPS and CORT Response on Novel Object Recognition.** No significant difference in duration of **A)** familiar or **B)** novel object investigation in males. No significant difference in **C)** velocity in males. No significant difference in duration of **D)** familiar or **E)** novel object investigation in females. No significant difference in **F)** velocity in females. All data were analyzed with two-way ANOVA. Mean+/- SEM, n=5/group.

**Supplementary Figure 5. Effects of SPS and CORT Response on Novel Object Discrimination Ratio.** No significant difference in novel object discrimination ratio in **A)** males or **B)** females. All data were analyzed with two-way ANOVA. Mean+/- SEM, n=5/group.

**Supplementary Figure 6. Effects of SPS and CORT Response on Social Interaction.** No significant difference in the duration of **A)** aggressive or **B)** submissive behaviors performed in males. No significant difference in **C)** rearing duration in males. No significant difference in the duration of **D)** aggressive or **E)** submissive behaviors performed in females. No significant difference in **F)** rearing duration in females. All data were analyzed with two-way ANOVA. Mean+/- SEM, n=5/group.

**SUPPLEMENTARY TABLE (as separate Excel file)**

**Supplementary Table 1: Detailed Statistical Results**

*Tab 1: Main Body Figures*. Statistical results for Figures 1-7, 9-10.

*Tab 2: Supplementary Figures*. Statistical results for Supplementary Figures 1-6.

*Tab 3: Correlation Matrix.* Statistical results for Figure 8.
